# Quantifying Type-Specific Reproduction Numbers for Nosocomial Pathogens: Evidence for Heightened Transmission of an Asian Sequence Type 239 MRSA Clone

**DOI:** 10.1371/journal.pcbi.1002454

**Published:** 2012-04-12

**Authors:** Ben S. Cooper, Theodore Kypraios, Rahul Batra, Duncan Wyncoll, Olga Tosas, Jonathan D. Edgeworth

**Affiliations:** 1 Centre for Clinical Vaccinology and Tropical Medicine, Nuffield Department of Clinical Medicine, University of Oxford, Oxford, United Kingdom; 2 Faculty of Tropical Medicine, Mahidol University, Bangkok, Thailand; 3 School of Mathematical Sciences, University of Nottingham, Nottingham, United Kingdom; 4 Centre for Clinical Infection and Diagnostics Research, Department of Infectious Diseases, King's College London and Guy's and St. Thomas' NHS Foundation Trust, London, United Kingdom; 5 The Intensive Care Unit, Guys and St. Thomas National Health Service Foundation Trust, London, United Kingdom; Princeton University, United States of America

## Abstract

An important determinant of a pathogen's success is the rate at which it is transmitted from infected to susceptible hosts. Although there are anecdotal reports that methicillin-resistant *Staphylococcus aureus* (MRSA) clones vary in their transmissibility in hospital settings, attempts to quantify such variation are lacking for common subtypes, as are methods for addressing this question using routinely-collected MRSA screening data in endemic settings. Here we present a method to quantify the time-varying transmissibility of different subtypes of common bacterial nosocomial pathogens using routine surveillance data. The method adapts approaches for estimating reproduction numbers based on the probabilistic reconstruction of epidemic trees, but uses relative hazards rather than serial intervals to assign probabilities to different sources for observed transmission events. The method is applied to data collected as part of a retrospective observational study of a concurrent MRSA outbreak in the United Kingdom with dominant endemic MRSA clones (ST22 and ST36) and an Asian ST239 MRSA strain (ST239-TW) in two linked adult intensive care units, and compared with an approach based on a fully parametric transmission model. The results provide support for the hypothesis that the clones responded differently to an infection control measure based on the use of topical antiseptics, which was more effective at reducing transmission of endemic clones. They also suggest that in one of the two ICUs patients colonized or infected with the ST239-TW MRSA clone had consistently higher risks of transmitting MRSA to patients free of MRSA. These findings represent some of the first quantitative evidence of enhanced transmissibility of a pandemic MRSA lineage, and highlight the potential value of tailoring hospital infection control measures to specific pathogen subtypes.

## Author Summary

Different strains of hospital pathogens may differ in their ability to spread between patients and respond differently to control measures. Attempts to quantify such between-strain variation are lacking in high prevalence settings. We analysed data from concurrent outbreaks with different MRSA strains in two adult intensive care units. MRSA is usually carried by patients asymptomatically, and most of our data came from routine screening swabs used to detect such carriage. We divided strains into two groups: common United Kingdom strains and strains from a type often found in Southeast Asia. We developed a new method to estimate how transmission changes over time and compared results with those from an adaptation of a previously described approach. An advantage of the new method is that it makes weaker assumptions about the process generating the data. The methods gave broadly similar results: the introduction of daily antiseptic bodywashes for all patients was the only intervention associated with a substantial fall in transmission, but this intervention was less effective for the Asian strain. This work should be useful for assessing the between-strain variation in the transmission of other hospital pathogens, and for assessing the impact of interventions on patient-to-patient transmission.

## Introduction

Methicillin-resistant *Staphylococcus aureus* (MRSA) is responsible for a high burden of morbidity and mortality worldwide [1]–[4]. While community-associated MRSA is becoming increasingly important globally [5], [6], in many countries, including the United Kingdom, MRSA remains predominantly a nosocomial pathogen [7], [8]. The dominant sequence type (ST) in Asia is ST239, and recent analysis of whole-genome sequence data has shown that this ST has distinct lineages in Asia, Europe and South America which probably share a European ancestor [9]–[11]. Little is known about what has enabled this ST to be so successful, or whether its propensity to transmit between hosts differs from other MRSA types in certain settings. A recent concurrent outbreak due to an ST239 MRSA strain (ST239-TW, subsequently referred to as TW) and the two dominant endemic UK MRSA types (ST22 and ST36, which we refer to as non-TW) in two linked adult intensive care units in a London teaching hospital provided a rare opportunity to compare the transmissibility of different MRSA types in the same clinical setting [12].

The transmissibility of a potentially emerging pathogen (the rate at which it spreads from an infected host to exposed susceptible hosts) is an important factor in determining its success and, in the case of an established pathogen, for estimating how effective interventions must be to bring an epidemic under control [13], [14]. Quantifying the degree to which strains of a nosocomial pathogen differ in their transmissibility in a particular setting could lead to a better understanding of why major clonal replacements occur. Measuring how such transmissibility changes in response to interventions would allow us to quantify the value of specific control measures, which may vary according to the strain [15]. This could lead to better resource use by allowing us to choose control measures appropriate for the specific strain. Such an analysis has greatest relevance for predominantly clonal organisms, such as *S. aureus*, where distinct lineages cocirculate over extended periods of time [10].

A fundamental measure of the overall transmission potential of a pathogen in a given setting is the basic reproduction number, 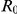. This is defined as the mean number of secondary cases generated by a typical case in a fully susceptible population [16], [17]. If the transmissibility of each infected host remains constant throughout its infectious period, and if each infected host has an equal chance of infecting each susceptible host, then 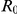 is simply the product of the mean rate at which an infected host generates secondary infections and the mean infectious period (provided the two are not correlated).

The self-sustaining chain reaction that constitutes a major epidemic is possible only if 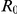 is greater than one. If it is less than one, although there may be some self-limiting chains of secondary transmission following the introduction of an index case (and quite large clusters become possible as 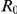 approaches one), this will not lead to a sustained increase in cases and, in a large population, only a small proportion of susceptible hosts will be infected [18]. An important related number is the net (or effective) reproduction number, 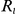. This is defined as the average number of secondary cases generated by a case infected at time 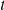, accounting for incomplete host susceptibility to infection and control measures in place. If 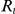 is greater than one at time 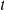, the epidemic will (on average) be growing. If 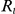 is less than one, it will be declining [16], [19].

These reproduction numbers are central to a mechanistic understanding of infectious disease epidemiology, and a number of methods for estimating them from different types of surveillance data have been devised [16], [19]–[23]. However, epidemics that predominantly affect hospitalized patients require some special considerations. First, unlike the community setting, the population of those exposed to infection changes rapidly over time as patients are admitted and discharged. Second, most common nosocomial pathogens are bacteria which can be carried asymptomatically over long periods, during which time colonized hosts may have several hospital admissions. This can give rise to distinctive dynamics: in addition to the usual explosive outbreaks, we also see epidemic patterns characterized by a sequence of self-limiting clusters of transmission which, over time, become more frequent and eventually coalesce into an exponentially growing epidemic [24], [25].

The concept of the single admission reproduction number, 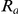, can help in the understanding of these features of hospital epidemics [25], [26]. 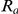 is defined as the mean number of secondary cases caused by a typical infectious patient during a single admission to a particular hospital or ward otherwise free of the pathogen. Necessarily, 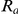 is less than or equal to 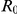. However, if 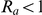 and 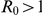 then every outbreak will be locally controlled in the short term, but, with repeated challenges to the hospital, long-term control failure will be inevitable. This results from the persistence of carriage following discharge which, over time, leads to a gradual increase in numbers colonized on admission. To account for changing numbers of susceptibles, we can also define a *net single admission reproduction number*, 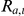. This is analogous to 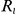 and represents the average number of secondary cases generated during a single hospital/ward admission where not everyone is necessarily susceptible.

Direct ascertainment of 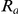 and 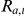 would be possible if we could reliably assess who infected whom during a hospital outbreak. In practice, even with detailed surveillance and molecular typing data, there is almost always considerable uncertainty about the true transmission tree. Instead, computationally-intensive approaches based on fitting mechanistic mathematical models to data which account for uncertainty in transmission routes and screening data represent the state-of-the art for analysing nosocomial transmission dynamics [27]–[32]. However, such approaches require detailed data on both susceptible and colonized or infected patients, and an assumption that temporal changes in the transmissibility can be described parametrically by some standard functional form (most commonly, piecewise constant). As currently implemented they do not allow direct estimates of the number of transmission events associated with each patient.

The aims of this paper are twofold: to describe a new approach (method 1) for estimating 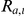 using hospital surveillance data; and to use it to analyse MRSA data from concurrent outbreaks with different MRSA types (TW and non-TW) in two linked adult intensive care units (ICUs). The method is simple to use and enables us to track how 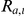 changes over time without the assumption that changes in transmissibility follow a fixed functional form, and without requiring data on susceptible patients. The method extends techniques for the probabilistic reconstruction of epidemic trees developed for analyzing foot and mouth disease and SARS data [21], [33]–[35]. We contrast results using this approach with that from a fully parametric mechanistic model (method 2), which represents an adaptation of previously described parametric models for nosocomial infection to a multistrain system [31], [32]. This second approach allows 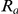 to be estimated. It requires more detailed data and stronger assumptions, but allows us to explicitly test hypotheses about how transmissibility is affected by interventions, and how it varies between different wards and subtypes of MRSA. While between-clone and between-ward differences in single admission effective reproduction numbers, 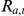, calculated using method 1 may be caused by differences in transmissibility, number of susceptibles, and lengths of stays, with method 2 we assume all MRSA positive patients have the same length of stay distribution and explicitly adjust for different numbers of susceptible patients when calculating 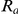.

## Results

### Probabilistic tree reconstruction (method 1)

**Figure 1:**
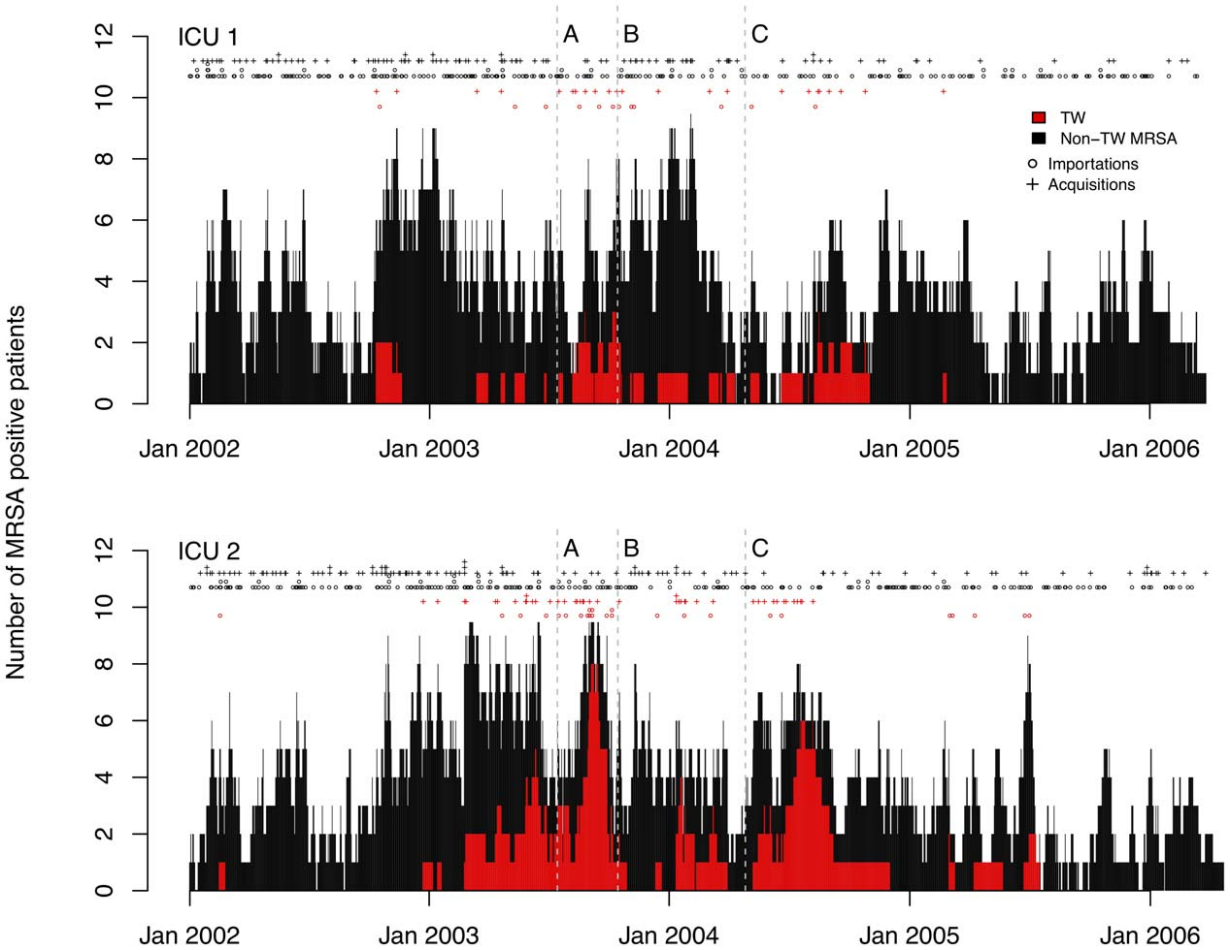
Incidence and prevalence of TW and non-TW MRSA strains on ICU 1 and ICU 2. Red and black filled areas indicate the number of patients known to be colonized or infected with TW and non-TW MRSA on each ward at each time point (assuming MRSA is not cleared during the ward stay). Vertical broken lines indicate the timing of interventions A, B and C. Symbols above each graph indicate the number of MRSA acquisitions and importations each day under baseline assumptions (see protocol S1 and table S1 in supporting information for details of assumptions and corresponding numbers under alternative assumptions).

Under baseline assumptions, on ICU1 there were 282 MRSA importation events (episodes where patients were assumed to be MRSA positive when admitted to the ICU) and 132 acquisition events. These comprised of 12 importations and 23 acquisitions with TW MRSA and 270 importations and 109 acquisitions with non-TW MRSA. On ICU2 there were 285 importations (25 with TW) and 166 acquisitions (43 with TW) (figure 1). Importations with non-TW to the respective ICUs decreased from 0.20 and 0.19 per day in phase 1 to 0.11 and 0.13 per day in phase 4. In contrast, importations with TW MRSA peaked in phase 2 in both ICUs (at 0.03 and 0.12 per day) and were at or below 0.01 per day in phases 1 and 4. Amongst patients who were MRSA positive on admission the median length of stay was 12 days (inter quartile range [IQR]4, 18) for TW-positive patients and 6 days (IQR 3, 13) for non-TW patients (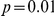, Wilcoxon rank sum test with continuity correction). For patients who acquired MRSA the corresponding numbers were 26.5 (13.25, 42.5) for TW patients and 19 (12, 29.25) for non-TW patients (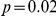). There was no evidence that length of stay differed by ward (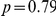), or by study phase (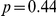, Kruskal-Wallis rank sum test).

**Figure 2:**
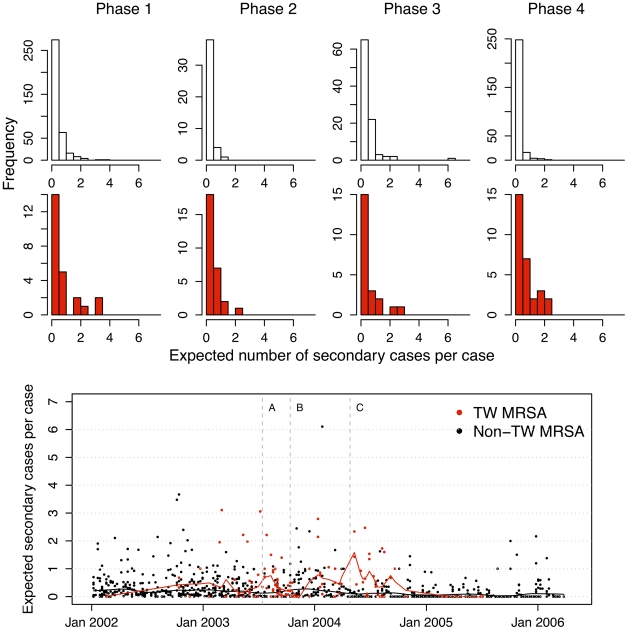
Expected numbers of secondary cases per case. Top panel shows histograms for expected numbers of secondary cases resulting from each case on the two ICUs (combined) in each of the four study phases (red is TW MRSA, white is non-TW) as calculated using method 1. The scatterplot (bottom panel) shows the same data, plotted according to the date MRSA was first isolated. Dates of interventions A, B and C are indicated by broken vertical lines. Smoothed trend lines (lowess smoothing where 10% of the points influence the smoothed value) are also shown.

Over the study period (January 2002 to April 2006) there were three interventions (referred to as A, B and C) and these define four study phases. Estimated net single admission case reproduction numbers (expected number of secondary cases per case during a single ward admission) associated with each MRSA-positive patient episode are shown in figure 2 (bottom panel) together with histograms of case reproduction numbers for each ward and study phase (top panel). These highlight wide between-patient variability which decreases in the second half of phase 4 when transmission is reduced and the TW clone is eliminated. While most patients have a very low expected number of secondary cases, 22 out of 103 patients (21%) with TW MRSA are expected to transmit to at least one other patient. Corresponding numbers for non-TW MRSA are 40 out of 762 (4%). This proportion was consistently higher for TW MRSA in all four study phases: 25, 11, 18 and 31% versus 7, 0, 9 and 2% for non-TW MRSA.

**Figure 3:**
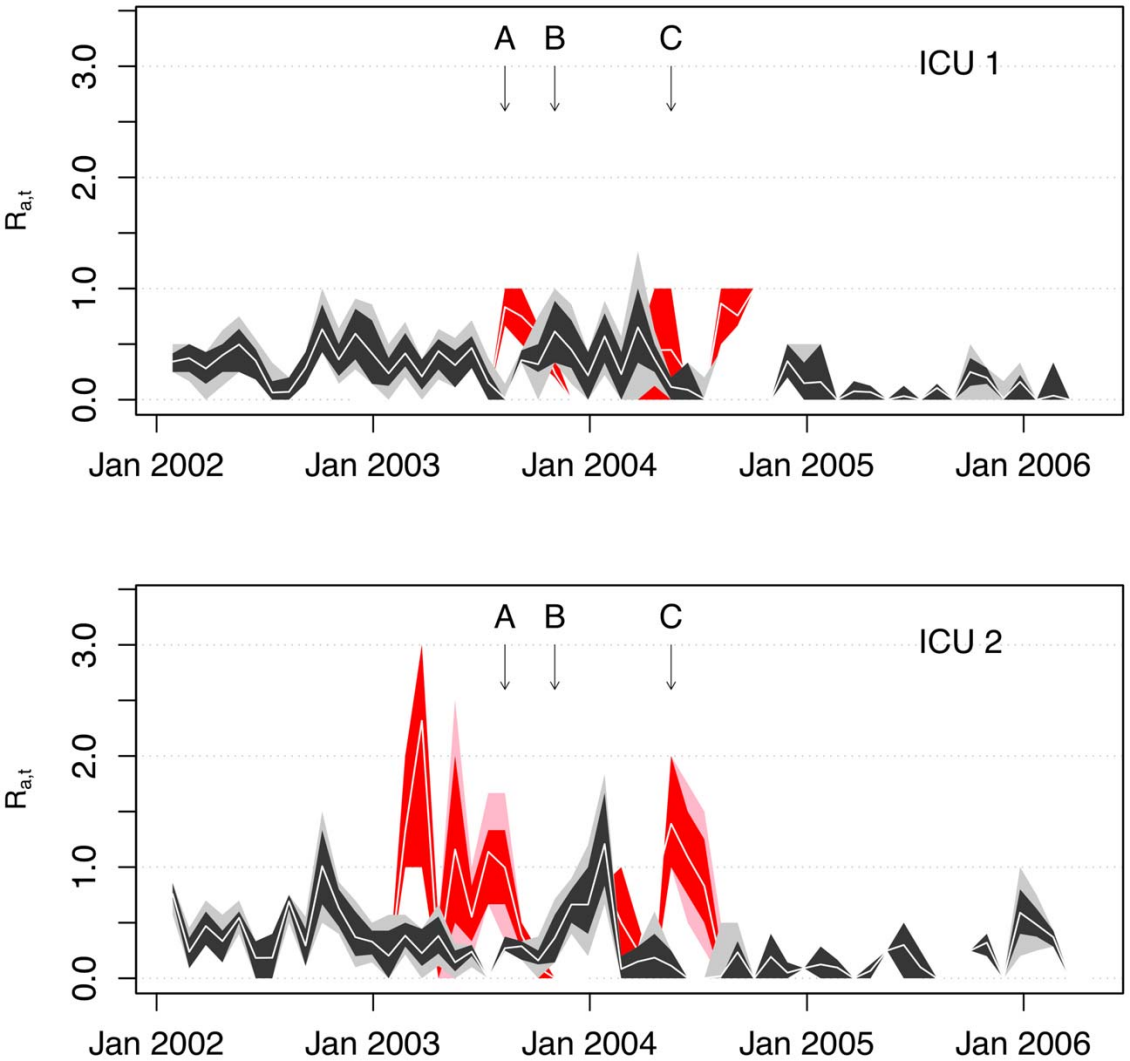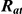 **estimates for MRSA types TW and non-TW obtained using method 1.** Estimates of net single admission reproduction numbers (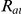) for TW clones (red) and non-TW clones (black) are shown in terms of the point estimate (central white line), 80% CIs (dark shaded region) and 95% CI (light shaded region) for each four-week averaged reproduction number obtained by averaging individual patient reproduction numbers according to the ICU admission date. Interventions A, B and C are indicated by vertical arrows.

Aggregating these reproduction numbers into four-week intervals highlights the temporal trends, differences between wards and impact of interventions (figure 3). In ICU1 these suggest similar patterns of transmission for the different MRSA types for the period prior to intervention C (a surface antiseptic protocol). In contrast, there were marked differences between MRSA types in ICU2 and throughout the study period the four-week averaged reproduction numbers for the TW clone usually exceeded those for non-TW clones when both types were present. These differences are also seen when reproduction numbers are averaged over study phases (table 1); the TW clone had a higher reproduction number than the non-TW MRSA in each phase in ICU2 but not in ICU1. Reproduction numbers for TW MRSA were also more volatile than those for non-TW MRSA in ICU2. There was evidence from both units to suggest differences between the MRSA types in their response to infection control interventions: while the net reproduction number of non-TW MRSA fell to a low level following intervention C in both ICUs, this was not the case for the TW clone which continued to transmit for several months at pre-intervention levels. Eventually, the TW outbreak came to an end after all patients with TW MRSA were treated empirically with systemic antibiotics (linezolid) from 1st September 2004 [12], [15]. After this intervention, although patients with TW MRSA continued to be imported into the ICUs, only three isolated apparent transmission events occurred (figure 1). When reproduction numbers for the two ICUs combined were estimated (allowing for cross transmission between ICUs) the results suggested the reproduction number of the TW clone was consistently higher than that for the non-TW MRSA and varied little throughout the study period (table 1). Reproduction numbers for the non-TW clones, in contrast, fell in phase 2 and 4. These results were not highly sensitive to the assumed strength of coupling between the two ICUs or to the MRSA acquisition assumptions (supplementary table S2).

**Table 1:** Estimated ward-level reproduction numbers (s.e.) for TW and non-TW MRSA clones using method 1.

		Phases			
		1	2	3	4
ICU1	TW MRSA	0.14 (0.10)	0.65 (0.17)	0.14 (0.08)	0.54 (0.22)
ICU1	Non-TW MRSA	0.36 (0.03)	0.31 (0.08)	0.41 (0.07)	0.14 (0.03)
ICU2	TW MRSA	0.73 (0.19)	0.41 (0.11)	0.58 (0.22)	0.58 (0.17)
ICU2	Non-TW MRSA	0.40 (0.04)	0.21 (0.06)	0.52 (0.15)	0.17 (0.03)
Combined ICUs	TW MRSA	0.71 (0.17)	0.50 (0.09)	0.56 (0.17)	0.64 (0.15)
Combined ICUs	Non-TW MRSA	0.38 (0.02)	0.20 (0.03)	0.47 (0.08)	0.15 (0.02)

Phase-specific estimates of ward-level reproduction numbers for TW MRSA and Non-TW MRSA derived using Method 1.

### Maximum likelihood (method 2)

Results from method 2 showed broad agreement with these findings, but in contrast to method 1 made *a priori* assumptions about the timing of the changes in transmissibility (tables 2–3, figure S1). On both wards, averaging over all phases, there was about a 1 in 400 chance of a given susceptible patient acquiring MRSA from a particular MRSA-positive patient on a particular day (table 2). When estimates of daily transmission probabilities from a single MRSA positive patient were constrained to take the same values for the TW and non-TW clones but were allowed to vary by ward and study phase, we found clinically significant variation between the four study phases in both ICUs (table 2). In particular, while estimates were similar in phases 1 to 3, there was a marked reduction in phase 4. There was no strong evidence that these joint estimates (for all MRSA clones) varied by ICU in any of the study phases (table 2). These findings were robust to the assumptions made about acquisition events and times (supplementary table S3).

**Table 2:** 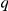
 estimates for TW and non-TW combined.

	Phases					P-value^1^
	1	2	3	4	All phases	
ICU1	0.0032 (0.0025, 0.0040)	0.0030 (0.0017, 0.0054)	0.0032 (0.0022, 0.0046)	0.0012 (0.0008, 0.0017)	0.0024 (0.0020, 0.0028)	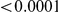
ICU2	0.0036 (0.0029, 0.0040)	0.0033 (0.0020, 0.0056)	0.0037 (0.0025, 0.0054)	0.0016 (0.0012, 0.0022)	0.0028 (0.0024, 0.0032)	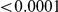
Combined	0.0034 (0.0029, 0.0040)	0.0032 (0.0022, 0.0047)	0.0034 (0.0026, 0.0044)	0.0014 (0.0011, 0.0018)	0.0026 (0.0023, 0.0029)	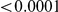
P-value^2^	0.17	0.43	0.82	0.54	0.23	

Phase-specific estimates for the daily probability of a susceptible patient acquiring MRSA from an MRSA positive patient in the same ward, for ICU 1 and ICU 2 (without distinguishing between TW and non-TW strains). In the *Combined* row, the estimates are constrained to be the same in both wards, and the *All phases* column constrains the estimates to be the same in the four phases.

1 P-values test the null hypothesis that transmission does not vary between study phase (likelihood ratio test, df = 3).

2 P-values test the null hypothesis that transmission in the current phase does not differ between wards (likelihood ratio test, df = 1).

**Table 3:** Estimates of the daily transmission probability (q) from one exposed to one susceptible patient.

Complete Bacterial Interference							
		Phases					P-value^1^
		1	2	3	4	All phases	
ICU1	TW MRSA	0.0010 (0.0002,0.0069)	0.0030(0.0011,0.0084)	0.0001(0.0000,0.0398)	0.0027(0.0011,0.0064)	0.0020(0.0011,0.0037)	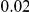
	Non-TW MRSA	0.0031(0.0025,0.0040)	0.0011(0.0003,0.0036)	0.0030(0.0020,0.0045)	0.0009(0.0006,0.0014)	0.0021(0.0017,0.0025)	
ICU2	TW MRSA	0.0040(0.0022,0.0074)	0.0037 (0.0019,0.0071)	0.0041(0.0019,0.0091)	0.0021 (0.0011,0.0037)	0.0031(0.0022,0.0042)	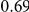
	Non-TW MRSA	0.0033(0.0027,0.0042)	0.0026(0.0011,0.0063)	0.0030(0.0019,0.0048)	0.0014 (0.0009,0.0020)	0.0025(0.0021,0.0030)	
Both	TW MRSA	0.0031(0.0017,0.0056)	0.0035(0.0021,0.0061)	0.0025(0.0011,0.0056)	0.0022 (0.0014,0.0036)	0.0027(0.0021,0.0037)	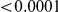
	Non-TW MRSA	0.0032(0.0027,0.0038)	0.0018(0.0009,0.0037)	0.0030(0.0022,0.0041)	0.0011 (0.0008,0.0015)	0.0023(0.0020,0.0026)	
P-value^2^		0.94	0.14	0.69	0.03	0.28	

Estimates of the daily transmission probability (q) from one exposed to one susceptible patient.

1 P-values test the null hypothesis that transmission varies between study phases but not MRSA types against the alternative that it varies between study phases and MRSA types (likelihood ratio test, df = 4).

2 P-values test the null hypothesis that transmission in the study phase does not differ between TW and Non-TW MRSA using combined data from both wards (likelihood ratio test, df = 1).

Extending this analysis to allow transmission probabilities to vary with the MRSA type enabled differences between MRSA clones and wards to be quantified (table 3) and allowed hypothesis tests about whether the daily transmission probabilities differed between strains (thus allowing us to test whether the observed differences in transmissibility found using method 1 could be entirely explained by the longer length of stay of the TW patients). In ICU1 no consistent differences were seen. In ICU2 the TW clone had a higher daily transmission probability to susceptible patients in each of the four study phases under the baseline assumption of complete bacterial interference, though confidence intervals were wide and showed considerable overlap. Differences between TW and non-TW MRSA transmission probabilities reached statistical significance at the 5% level for both wards combined and for ICU1, but not for ICU2 alone, and in only one of the four phases (phase 4) using combined data from both ICUs. This phase corresponded to the introduction of the surface antiseptic bodywash protocol, which was associated with a more than halving of the transmission probability from a patient with non-TW MRSA compared to earlier phases. The fall in the transmission probability for TW MRSA in phase 4 was smaller, and likely to be confounded by the use of linezolid for TW carriers in this phase. Large differences in transmission probabilities for the two MRSA types were also seen in phase 2 (corresponding to the introduction of hand hygiene promotion), but in this case confidence intervals were wider reflecting the short duration of this phase. In other phases differences between TW and non-TW estimates were much smaller. The magnitude of the differences depended on which patients were assumed to be susceptible. Under the baseline assumption that patients colonized with one strain were not susceptible to acquiring another (complete bacterial interference) the differences were larger than in the sensitivity analysis where no bacterial interference was assumed (table 3). This can be explained by the higher prevalence of non-TW MRSA clones; under the assumption of no bacterial interference all non-TW MRSA positive patients would be considered susceptible to infection or colonisation with TW MRSA and vice versa. Changing from complete interference to no interference therefore results in a greater increase in the number of susceptibles available for the TW clones to infect than it does for the non-TW clones. To accommodate these changes, a larger reduction in the daily transmission probability for TW clones is required. Overall, combining data from both wards, the TW clone was estimated to have a daily transmission probability that was between 63 and 100% higher than the non-TW clones in phase 4, and between 53 and 94% higher in phase 2 (the lower numbers corresponding to the no bacterial interference assumption) though the differences only reached significance at the 5% level in phase 4 and only under baseline interference assumptions. Transmission probabilities were broadly similar in the two other study phases. Estimates of the single-admission reproduction number (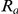) from the model without background transmission (and assuming TW and non-TW patients have the same length of stay distribution) are reported in the supplementary material (figure S1). Results were robust to the assumptions made about the number and timing of MRSA acquisition events (supplementary table S4), but fitting a more complex model allowing patient-to-patient transmission and transmission from background sources suggested that the relative importance of patient-to-patient and background transmission could not be reliably identified in such hyperendemic settings without additional data (supplementary table S5).

## Discussion

Common bacterial nosocomial pathogens have distinct dynamics from typical community pathogens and call for different analytical approaches. Important features of hospital epidemics with such organisms include: i) a host population that changes rapidly over time in comparison with the timescale of epidemic dynamics; ii) a high proportion of infected (or colonized) hosts who are already infected when they enter the population (the hospital or ward); iii) a dominant role for asymptomatic infection so infected hosts can usually only be identified using screening swabs, leading to large uncertainty in the timing of transmission events; iv) a lack of a well-defined serial interval or generation time (since asymptomatic carriage can persist for months or years, but transmission is only intermittently observed during hospital admissions). The probabilistic tree reconstruction approach described above (method 1) overcame these limitations by using a hazards-based approach applied to patient screening data to assign probabilities to potential source patients for observed acquisition events. Using hazards in this way to reconstruct epidemic trees and estimate reproduction numbers appears to have first been suggested by Kenah *et al* [36]. Results using this method were supplemented with a maximum likelihood approach (method 2) where the timing of cross-infection events was assumed to be known but which allowed estimation of the daily transmission probability, enabling us to study effects related to study phase and MRSA type while controlling for differences in length of stay.

These methods were applied to data from two adjacent general ICUs in which admission and weekly MRSA screens and culture results from clinical samples identified patients admitted with and acquiring MRSA over a four year period. During that time there was sustained transmission with endemic MRSA and a newly introduced TW variant.

Both analytical methods supported the hypothesis that intervention C (the surface antiseptic protocol) was associated with a sustained reduction in MRSA transmission, and both indicated a reduced effect for the TW clone. Both methods gave point estimates that indicated elevated transmission of TW MRSA compared with endemic strains in all four study phases in ICU2 but not ICU1. There were, however, some differences: the ward-level reproduction numbers (method 1) tended to indicate greater increased transmission for the TW compared to non-TW MRSA than was seen using method 2. This reflects the fact that the two methods are quantifying different things: method 1 estimates secondary cases per case, which depends both on transmissibility and the length of ICU stay while carrying MRSA; method 2, in contrast, estimates only the daily transmission probability from one MRSA carrier to one susceptible patient. This will not be affected by length of stay. Indeed, there was some evidence that patients colonised with TW MRSA (particularly those colonised on ICU admission), had a longer length of stay than those colonised with non-TW MRSA. This may reflect the link between MRSA infection and excess length of stay in this cohort [37], and the increased virulence of the TW strain which was over four times more likely to cause blood stream infection in colonised patients compared to non-TW MRSA strains in the same ICUs [12]. Even in the absence of an increased rate of transmission to other patients, increased length of stay would lead to a higher single-admission reproduction number. It is possible that such differences in length of stay reflect underlying differences in the characteristics of patients most vulnerable to acquiring the different MRSA types. For example, because the TW outbreak was centred on the two ICUs, patients carrying TW on ICU admission might be more likely than patients carrying non-TW MRSA to have had recent ICU admissions. The TW clones showed a far broader range of antibiotic-resistance than endemic MRSA clones and have previously been shown to preferentially colonise vascular catheters but not carriage sites compared with endemic strains [12]. Taken together, these observations suggest that the TW MRSA could represent a phenotype particularly adapted to transmission in settings, such as ICUs, with high levels of antibiotic usage and patient catheterisation, perhaps at the expense of persistence outside these areas. There is some evidence that such adaptation results from both increased persistence in the ICU (perhaps by targeting long-stay patients, and causing infections that increase length of stay) and from an increased daily transmission probability (particularly in the presence of widespread antiseptic use). Caveats, of course, apply: differences in lengths of stays between TW and non-TW colonized/infected patients could be confounded by exposure history (the recent arrival of the TW clone rather than its biological properties may account for the different patient characteristics). Differences in daily transmission probabilities could also be subject to such confounding and could also have arisen by chance (in all phases – even phase 4, where the effect size was largest – confidence intervals were wide).

The mechanisms underlying variations in transmissibility of different MRSA (and *S. aureus*) strains are poorly understood. Reasons for the differences in the two ICUs are also unclear. Chance variation cannot be ruled out, as the formal investigation of transmission potential of different MRSA types was, in part, motivated by perceived differences in transmissibility (using the same data), and the usual limitations of *post hoc* analyses therefore apply. Also, although the analyses accounts for demographic stochasticity, there may also be important sources of environmental stochasticity which are not accounted for. It seems unlikely that the difference in TW transmission in the two ICUs can be explained by colonized staff: a universal staff screening programme failed to detect the TW clone during the outbreak [12]. Differences in infection control practice also seem unlikely but cannot be ruled out: the two wards share the same infection control policies and staff pool, with medical and nursing staff rotating between units at 3–6 monthly intervals, though only physiotherapy, radiology and pharmacy staff worked across both units at the same time. It is possible that the built environment influences MRSA transmission. ICU2 was last refurbished in 1969, retaining a mixture of original materials including wood, and has much less open space, only eight sinks, and one side room, whereas ICU1 was refurbished in 1999 to an open plan configuration with better space utilization, 19 sinks and three side rooms. The reduced availability of sinks, side rooms and space to circulate may have adversely affected the ability to carry out infection control practice or cleaning, although it is unclear why this should only affect TW MRSA, which was not detected on environmental screening during the outbreak [12].

Despite anecdotal reports that some lineages of *S. aureus* strains have an enhanced epidemic potential in hospital settings [38], objective assessments of between-strain variation in transmissibility are largely lacking. Such variation is nonetheless to be expected given the large degree of phenotypic variation in different *S. aureus* and MRSA clones, and the dominance of a small number of MRSA lineages [39]. One of the few instances where the nosocomial transmission potential of different subtypes of the same nosocomial pathogen have been quantified comes from a comparison of the onward transmission from patients admitted to hospitals in the Netherlands carrying MRSA [40]. In this case, because MRSA introductions were infrequent (as MRSA prevalence in hospitals in the Netherlands is below 1%) and contact tracing extensive, the secondary cases could be assigned to distinct clusters of transmission following identified introductions. This allowed the authors to use methods based on a branching process model to estimate the single admission reproduction number, 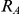 [41]. It was found that newly admitted ST398 MRSA strains (which are commonly associated with livestock production) had a greatly reduced propensity to spread compared with other MRSA sequence types, with an 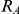 value (95% CI) of only 0.16 (0.04–0.40), about one sixth of the corresponding value for non-ST398 MRSA. The authors concluded that less stringent control measures were likely to be sufficient to control ST398 MRSA clones than those needed for non-ST398 MRSA types.

Such methods would not have been applicable for our data, and the first method used here to quantify the transmissibility of different strains (method 1) instead built on recent approaches to estimate reproduction numbers by probabilistically reconstructing epidemic trees. Such tree-reconstructions have used simple rule-based methods, for example assigning sources from a candidate list based on proximity data [33], more formal semi-parametric methods using partial likelihoods and assuming a known serial interval distribution [21], [34], and, most recently, semi-parametric hazard-based approaches [36]. Hazard-based approaches have some advantages over the first two methods: they avoid some of the arbitrary assumptions of the rule-based approaches, do not require knowledge of the serial interval distribution, and can avoid biases that arise from the fact that the serial interval distribution changes over the course of an epidemic. Advantages over approaches based on fitting a full transmission model include fewer assumptions, in particular with regard to the functional form of changes in the transmission potential over time. In this respect, tree reconstruction approaches have some similarities with other semi-parametric approaches that make use of survival analytical methods, such as the approach adopted by Wolkewitz *et al.*, who derived non-parametric estimates of a time-varying transmission rate changed over time using a Martingale-based method [42]. An important difference in the current approach is that we are specifically interested in estimating how the distribution of the number of secondary cases resulting from each case changes over time. The method 1 approach described here also makes relatively low demands for data (with no information required for patients who do not become colonized or infected), has a low computational burden, and can be easily adapted to cope with co-circulating subtypes as in the application here. This approach is appropriate when the daily probability of a patient acquiring MRSA is small, as in this case reconstructed epidemic trees will be approximately independent of this probability. This approximation is likely to be reasonable for all but the most explosive outbreaks. For example, using the exact formula we found that changing this probability from a baseline of 0.005 to 0.001 and 0.025 changed the estimated mean reproduction numbers for each phase and MRSA type by less than 3%.

Two assumptions underlying the analytical approaches used here are i) that new MRSA acquisitions can be explained by patient-to-patient spread within the units (which is likely to be mediated by contacts with transiently colonized healthcare workers) and ii) that risk of transmission increases in line with colonization pressure (the number of patients with MRSA on the ward). While these assumptions are supported by observational and quasi experimental studies [43], [44], it would be desirable to more rigorously challenge them. Unfortunately, unpublished simulation studies and analysis here with a more complex model allowing different transmission routes (table S5) both suggest that the ability to identify the relative importance of background and patient-to-patient transmission may be limited in hyper-endemic settings in the absence of more discriminatory typing data. The inability of our typing methods to reliably distinguish between non-TW MRSA types, or to identify genetic variants of the TW clone therefore represent important limitations of this work. High resolution genotyping data would enable more definitive assessments of who infects whom, and therefore allow us to quantify the risks of transmission of different MRSA subtypes in different wards at different times with greater certainty.

Figure 1 confirms that not all acquisition events can be explained by transmission from a known MRSA positive patient from the same ward. The combined-ICU analysis, allowing for between-ward transmission, is able to account for some MRSA acquisitions where no known source was present on the same ward, and this explains why combined ICU estimates of the reproduction number are sometimes outside the range of individual ICU estimates (table 1), but unknown MRSA sources are also likely to be present in the patient population [32]. A full model-based analysis using data augmentation (which estimates model parameters and latent parameters that represent “unobserved” - or augmented - data, typically using Markov chain Monte Carlo methods for fitting) could account for such unknown sources. Such an approach retains some important advantages for analysing typical surveillance data. These include the ability to account for imperfect swab sensitivity and for uncertainty in the number and timing of acquisition events, circumventing the need to make arbitrary assumptions about which patients were colonized on admission to a ward. In the present context such an analysis would allow us to explicitly account for the change in the screening protocol in November 2004. Since this involved screening more body sites, it is likely to have increased screening sensitivity and led to increased detection of MRSA, potentially biasing the estimated effect of intervention C. To date, however, no published work has adapted such approaches to cope with multiple co-circulating subtypes. The method 2 used here can be thought of as a simplified version of such an approach (in that it is based on a fully-specified mechanistic transmission model) but it avoids the complexities of data augmentation by assuming the epidemic process is perfectly observed. An important area for future work will be to extend data augmentation methods to cope with carriage of multiple types. Such approaches have been developed for the sequential carriage of community pathogen subtypes [45]. Addressing issues of co-colonisation with different subtypes may be particularly important for some nosocomial pathogens, and neglecting such effects is a potential source of bias. In our analysis here we considered two possibilities – complete bacterial interference (where one strain completely inhibits the acquisition of another), and no bacterial interference. The reality may lie somewhere between these two extremes. Such an analysis will be complicated by the fact that routinely-used laboratory methods are not well-suited to detecting the simultaneous carriage of multiple types [46], and sensitivity for detecting a second type will not, in general, be the same as sensitivity for detecting a single type.

## Methods

### Ethics statement

Ethical approval for this research was granted by the NHS National Research Ethics Service, South East Research Ethics Committee. All data were analyzed anonymously.

### Clinical data and infection control practice

Anonymised data from two 15-bed adult general intensive care units (ICU) within a 1050-bed teaching hospital in London, United Kingdom, were collected between 1st January 2002 and 30th April 2006 as described elsewhere [12], [15]. Dates of admission and discharge and MRSA culture results from screen and clinical samples were analysed for all 4,570 consecutive patient admissions to both ICUs. Infection control policies were in place including specifying hand hygiene between patient contacts and use of contact precautions for known MRSA colonized patients throughout. On this background three main new MRSA control interventions were introduced: intervention A (introduced on 15th July 2003) was an education campaign to promote hand hygiene and barrier nursing; intervention B (introduced on 15th October 2003) was isolation of known MRSA colonized patients in side rooms or in patient and nursing cohort pairs; intervention C (introduced on 26th April 2004) was a surface antiseptic protocol which included daily chlorhexidine bodywashes for known MRSA positive patients, and daily triclosan bodywashes for other patients. The three interventions defined four study phases for analysis: phase 1 from 1st January 2002 to 14th July 2003; phase 2 from 15th July to 14th October 2003; phase 3 from 15th October 2003 to 25th April 2004; and phase 4 from 26th April 2004 to 30th April 2006. Patients were swabbed for MRSA carriage on admission and every Monday morning. Swabs were taken from nose, axillae and perineum until 1st November 2004, when additional rectal and throat samples were included (a change associated with an approximate 30% increase in the proportion of patients identified as carriers on admission to ICU) [47]. Clinical samples were collected when infection was suspected. *S. aureus* colonies were identified using a combination of catalase positivity, Staphaurex (Remel Europe Ltd., Dartford, England) and/or salt mannite positivity with confirmation by a tube coagulase test. Methicillin resistance was determined by disc testing. Screen samples were identified using a selective mannitol broth technique [47].

TW MRSA was defined initially by its distinctive and extensive antimicrobial resistance pattern, sequence typing and microarray analysis [12]. More extensive typing of available admission and acquisition isolates has shown all antimicrobial resistance patterns defined TW isolates to belong to CC8/239 and non-TW isolates to be 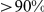 ST22 and ST36 [15]. When TW and non-TW MRSA isolates were recovered from the same patient, only the first type recovered was considered. This was, however, rare: two patients had both types recovered from pooled screening sites; nine had both types from sputum; and seven had both types from wounds. Thirteen patients had both types recovered from different sites. Further details of patient characteristics, interventions, swabbing sites and microbiological procedures have been described elsewhere [12], [15].

### Analysis

We analyse the data using two separate approaches which we refer to as method 1 and method 2. In both analyses we define a new MRSA acquisition to have occurred if a patient has a negative admission screening swab, a subsequent MRSA positive screen or clinical sample while in the ICU and more than 48 hours after being admitted to the ward, and no prior MRSA positive isolate in the 90 days preceding ICU admission. Patients with any MRSA positive samples taken within 48 hours of admission are assumed to be positive on admission (MRSA importations). A patient who is believed to be neither colonized nor infected on a given day is assumed to be susceptible to becoming colonized or infected by either MRSA type (see supplementary material for further details). In the first approach (method 1), which probabilistically reconstructs the epidemic tree, we assume that the acquisition occurred one or more days before the first positive screening swab. In the second approach (method 2) we assume a new acquisition to have occurred on day 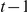 if a patient has his or her first MRSA positive swab on day 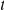, following a negative MRSA admission screening swab during the same ward admission.

We also assume i) that once MRSA positive, a patient remains so until ward discharge (hence no information from swab results after the first positive is used), and ii) MRSA-positive patients only become potential sources for transmission to other patients after their first positive swab, unless they are assumed to be positive on admission, in which case they are potential sources from their date of admission. For patients readmitted to one of the wards following ward discharge, we apply the same criteria that we use for first time admission to determine admission colonisation status. We use a time unit of one day, and take dates of admission and discharge to represent the first and last whole days of a patient admission.

#### Notation

We introduce the following notation: let 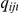 represent the daily probability of a single susceptible patient in ward 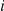 acquiring MRSA of type 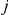 from a single patient on the same ward at day 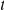 who is colonized or infected with MRSA type 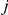. We also define the daily avoidance probability of acquiring MRSA, 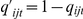.

Denote by 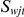, 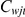, and 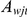 the number of patients on ward 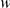 on day 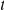 who are, respectively, susceptible to MRSA type 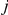, known to be colonized or infected with type 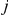, and found to be colonized or infected with type 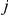 for the first time on day 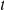 (having had a prior negative admission screening swab). Here we take 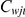 to be the number of patients on ward 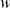 on day 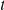 who have had at least one previous positive swab with MRSA type 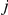 on or before day 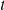. We take 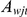 to be the number of patients on ward 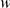 who have their first positive swab with MRSA of type 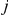 on day 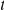. In our default method 2 analysis we take 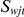 as the number of remaining patients (i.e. those with no prior MRSA positive swabs during the current episode) excluding those patients who are discharged on day 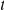 since we assume that acquisitions on the day of discharge would be not be detected. The tree reconstruction approach (method 1), in contrast, does not require knowledge of 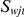 to estimate reproduction numbers.

In the application we consider here there are two wards and we define two subtypes (TW and non-TW), so both 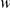 and 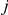 can take values 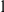 or 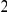. We also define 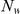 to be the total number of patient episodes on ward 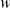 over the study period for which there was at least one MRSA positive swab and 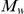 to be the total number of new MRSA acquisitions on ward 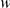 over the study period, i.e. 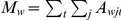.

#### Method 1: Reconstruction of the epidemic tree

The tree-reconstruction approach calculates the probability that each observed new MRSA acquisition was acquired from each of the other MRSA positive patients in one of the two ICUs. In this approach we condition on the probabilities 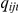 and assume that all new acquisitions were acquired from a known patient source. A scaling factor, 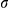, specifies the reduction in the daily risk of transmission from an MRSA positive patient in one ward to an MRSA negative patient in a different ward. We explore the sensitivity of the results to both the 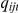 and the 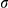 values.

Let 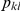 represent the conditional probability that patient 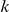 acquired MRSA from patient 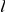 given that patient 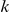 acquired MRSA from one of the other 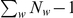 MRSA positive patients. We calculate the elements 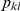 of the 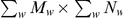 matrix 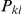 as follows. Define 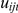 to be the probability that a susceptible patient on ward 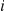 at time 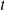 escapes cross-infection from one of the 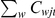 MRSA type 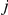 positive patients in the ICUs on that day. Therefore 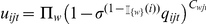 where 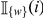 is an indicator function that equals one when 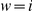 and zero otherwise. Now consider a single patient 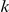 on ward 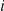 who is free of MRSA on admission and whose first and last days on the ward are 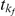 and 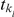. The probability that this patient is free of MRSA type 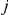 at the end of day 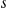 (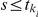) is
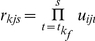
and the probability that this patient acquires MRSA of type 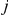 from patient 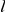 on day 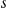 is given by
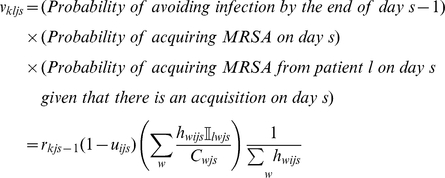
where the 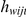 terms represent the hazards of transmission of MRSA type 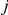 from patients in ward 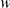 at time 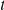 to a patient in ward 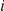 and 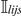 is an indicator function that takes the value 1 if patient 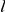 is present on ward 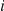 and MRSA type 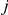 positive on day 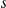 and 0 otherwise. These hazards can be expressed in terms of the probabilities 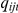 as 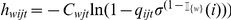, which is approximated by 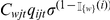 when 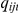 is small, as will usually be the case.

The conditional probabilities, 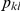, which represent the probability that patient 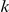 was infected by patient 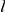 given that patient 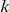 was infected by one other patient, are then given by the following expression, which is approximately independent of 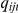 when 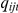 is small:
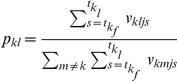
Here 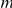 indexes all the patients who could potentially have infected patient 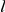. The net single admission reproduction number for patient episode 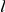 (i.e. the expected number of secondary cases resulting from this episode) is then given by
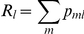
and corresponding reproduction numbers for a given time period are obtained by averaging over these patient reproduction numbers for all patient episodes starting in the given period. Associated confidence intervals are derived by simulation, by repeatedly drawing the source of infection for each of the 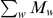 new infections from a multinomial distribution with probability vectors given by the rows of 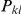. All confidence intervals reported for reproduction numbers are based on the quantiles of 1000 such simulations.

By default, when analysing both wards together, we assume minimal cross-infection between the wards (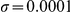), though we also consider the opposite extreme (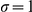), representing complete ward mixing. We report results here where 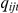 is fixed at 0.005, though also describe results of sensitivity analyses with values of 0.001 and 0.025.

#### Method 2: A likelihood-based approach

The second approach estimates the probabilities 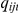 using maximum likelihood estimation (MLE). With two MRSA types, we assume that patients susceptible to type 1 are also susceptible to type 2, so 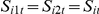. This implicitly assumes complete bacterial interference (i.e. that colonisation with one type of MRSA prevents acquisition of another type one or more days after the first acquisition [48]). As a sensitivity analysis we also considered the other extreme: complete lack of bacterial interference, so that a patient colonized with one MRSA type had the same daily risk of acquiring a different subtype as an uncolonized patient. With the additional assumption that new MRSA acquisitions occur the day before they are detected, the log likelihood of the new MRSA acquisition data in ward 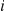 on day 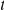 is given by:
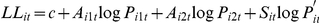
where 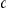 is a constant and

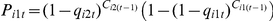

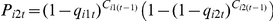

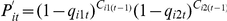

Here 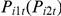 is given by the product of the probability of not acquiring MRSA type 2(1) on day 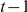 and the probability of acquiring type 1(2). 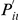 is the product of the probabilities of not acquiring either MRSA type. For completeness we could add a term to represent acquisition of both types on the same day. In practice we had no such observations.

The overall log-likelihood is then given by the sum of 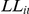 terms over 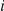 (the two wards) and is maximized using unconstrained optimization with the Nelder-Mead algorithm [49] as implemented in the function optim in R version 2.11.1 (www.project-r.org). Approximate 95% confidence intervals (CIs) were derived by inverting the square matrix of second-order partial derivatives of the loglikelihood function, i.e. the Hessian.

In practice, with method 2 rather than estimating separate 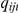 terms for every day 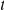 (which would over-parameterize the model), we apply constraints. We consider constraints where i) 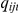 terms from the same ward and same study phase are required to take the same value; ii) 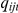 are the same across all time periods within each ward; iii) 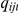 terms from the same study phase are constrained to take the same value and do not vary across study wards; and iv) 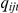 terms are the same for different MRSA types, 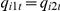. These constraints imply a series of nested models, and we apply likelihood ratio tests to determine whether there is evidence to reject the hypotheses that these equality constraints represent.

To obtain estimates of 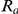 requires consideration of the length of stay distribution. To simplify matters we ignore any potential additional length of ICU stay caused by infection, but account for the facts that the longer a susceptible patient stays the greater the risk of acquiring MRSA, and the longer an MRSA-positive patient stays the greater the expected number of secondary transmission events they will cause. In simple models it is often assumed that there is constant hazard of hospital discharge and the length of stay distribution is exponential. In such cases the risk of a patient acquiring MRSA is unrelated to the subsequent length of stay and 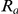 is trivially calculated as the product of mean length of stay, the mean number of exposed patients on the ward, and the daily probability of transmission from a single source to a single exposed patient. In practice, the hazard of ICU discharge is likely to be dependent on the day of stay, and typically decreases with increasing day of stay. In this case, the patients at greatest risk of acquiring MRSA (i.e. those who have stayed longest on the ward) will also have longer expected future stays and will therefore tend to cause more secondary infections. To account for this we partition patients into groups defined by the number of ICU days a randomly-selected patient on the ward would stay after becoming MRSA positive (assuming no additional stay due to MRSA). To do this we use the empirical length of stay distribution and calculate the probability, 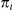, that a randomly selected patient on the ICU is a member of group 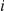. This is given by 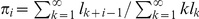, where 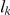 is the probability that a newly admitted patient stays for 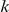 days. Assuming an average of 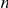 exposed patients per day on the ward, and that each patient has a daily probability, 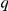, of acquiring MRSA from a single MRSA positive patient on the ward, an MRSA patient in group 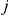 will, on average, transmit MRSA to 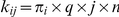 patients in group 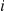 (ignoring saturation effects, which will be negligible for sufficiently small 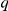). These 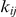 values are the elements of the next generation matrix. The dominant eigenvalue of this matrix gives the reproduction number, 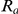 [20].

In contrast to method 1, which excludes susceptible patients from the analysis and enables estimates only of the net (or effective) reproduction number, this method accounts for susceptibles in the model and therefore allows us to estimate the single-admission reproduction number (the transmission potential of an MRSA positive patient in an otherwise fully susceptibility ward). This will be greater than or equal to the net single-admission reproduction number, and determines the threshold epidemic behaviour [25]. A further difference is that this method allows the transmission potential of an MRSA positive patient to change over time, according to the current study phase. In contrast, method 1 makes no explicit assumptions about the timing of changes in the transmission potential, and net reproduction numbers for a particular time period relate to the transmission potential of patients admitted to the ward during that time period even though the actual transmission events may occur at a later time.

Both methods 1 and 2 assume that MRSA acquisition events occur as a result of patient-to-patient transmission from known carriers and exclude observations where there are no potential source patients. An additional sensitivity analysis therefore extended method 2 by allowing for both patient-to-patient transmission and background transmission (for example, from colonized staff or persistent environmental contamination). If the daily probability of a susceptible patient on ward 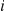 at time 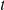 acquiring strain j from such background sources is 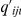, then the 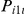 term above is replaced by 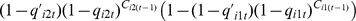 in the new model, with similar changes for other terms. When applying this model, 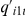 was assumed to vary by ward, MRSA type and study phase, but to remain constant within in a phase.

## Supporting Information

Figure S1
**Single admission reproduction numbers (** 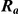 **) estimated using method 2.** Estimates (95% CIs) of the ward-level reproduction number, 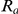, according to study phase, MRSA type and ward obtained using method 2 and assuming complete bacterial interference and no interaction between ICU 1 and ICU 2.(PDF)

Protocol S1 **Protocol for defining MRSA importation and acquisition events.**
(PDF)

Table S1 **TW and non-TW MRSA importation and acquisition events under different assumptions.** The baseline assumption classifies all episodes where MRSA was recovered from an isolate taken within 48 hours of admission as importations. The SA1 assumption uses a 24 hour cutoff instead. See protocol S1 in supporting material for full details of baseline and SA1 assumptions.
(PDF)

Table S2 **Estimated ward-level reproduction numbers (s.e.) for TW and non-TW MRSA clones under alternative assumptions.** Phase-specific estimates of ward-level reproduction numbers for TW MRSA and Non-TW MRSA derived using Method 1 under baseline assumptions with perfect ward coupling (applies to combined ICU estimates only) and under SA1 assumptions (see protocol S1 in supporting material for details of baseline and SA1 assumptions).
(PDF)

Table S3

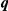
 **estimates for TW and non-TW combined under SA1 and SA2 assumptions.** Sensitivity analysis for phase-specific estimates for 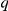, the daily probability of a susceptible patient acquiring MRSA from an MRSA positive patient in the same ward, for ICU 1 and ICU 2 (without distinguishing between TW and non-TW strains). In the *Combined* row, the estimates are constrained to be the same in both wards, and the *All phases* column constrains the estimates to be the same in the four phases. See protocol S1 in supporting material for details of the SA1 and SA2 assumptions used in the sensitivity analyses. 1 P-values test the null hypothesis that transmission does not vary between study phase (likelihood ratio test, df = 3). 2 P-values test the null hypothesis that transmission in the current phase does not differ between wards (likelihood ratio test, df = 1).(PDF)

Table S4 **Estimates of the daily transmission probability (q) from one exposed to one susceptible patient under SA1 and SA2 assumptions.** Estimates of the daily transmission probability (q) from one exposed to one susceptible patient under assumptions SA1 and SA2. See protocol S1 in supporting material for details of the SA1 and SA2 assumptions used in thesse sensitivity analyses. 1. P-values test the null hypothesis that transmission varies between study phases but not MRSA types against the alternative that it varies between study phases and MRSA types (likelihood ratio test, df = 4). 2 P-values test the null hypothesis that transmission in the study phase does not differ between TW and Non-TW MRSA using combined data from both wards (likelihood ratio test, df = 1).
(PDF)

Table S5 **Estimates of the daily transmission probability (q) from one exposed to one susceptible patient and from background transmission sources.** ‘Patient to patient’ estimates corresponds to the daily transmission probability (q) from one exposed to one susceptible patient. Background estimates corresponds to the daily probability of acquisition from background sources (such as environmental contamination). This probability is assumed to remain constant within each phase for each of the two MRSA types. In some cases confidence intervals could not be estimated for numerical reasons, while in others the very wide confidence intervals indicate that parameters are weakly identifiable. For both ICUs we compared the model with background and patient-to-patient transmission (with both phase and MRSA-type specific parameters) with nested models with only background transmission (but still with both phase and MRSA-type specific parameters) using a likelihood ratio test based on the chi-squared distribution with eight degrees of freedom. The results gave strong evidence to prefer the more complex model in the case of ICU2 (p = 0.008), but no evidence to prefer it in the case of ICU 1 (p = 0.70).
(PDF)

## References

[1] de Kraker ME, Davey PG, Grundmann H, BURDEN study group (2011). Mortality and hospital stay associated with resistant Staphylococcus aureus and Escherichia coli bacteremia: estimating the burden of antibiotic resistance in europe.. PLoS Med.

[2] Gotuzzo E (2010). Current status and recommendations on methicillin-resistant Staphylococcus aureus infection in Latin America.. Braz J Infect Dis.

[3] Klevens RM, Morrison MA, Nadle J, Petit S, Gershman K (2007). Invasive methicillin-resistant Staphylococcus aureus infections in the United States.. JAMA.

[4] Nickerson EK, Wuthiekanun V, Day NP, Chaowagul W, Peacock SJ (2006). Meticillin-resistant Staphylococcus aureus in rural Asia.. Lancet Infect Dis.

[5] Vandenesch F, Naimi T, Enright MC, Lina G, Nimmo GR (2003). Community-acquired methicillin-resistant Staphylococcus aureus carrying Panton-Valentine leukocidin genes: worldwide emergence.. Emerg Infect Dis.

[6] Nickerson EK, Wuthiekanun V, Kumar V, Amornchai P, Wongdeethai N (2011). Emergence of community-associated methicillin-resistant Staphylococcus aureus carriage in children in Cambodia.. Am J Trop Med Hyg.

[7] Jeyaratnam D, Reid C, Kearns A, Klein J (2006). Community associated MRSA: an alert to pae-diatricians.. Arch Dis Child.

[8] Johnson AP, Sharland M, Goodall CM, Blackburn R, Kearns AM (2010). Enhanced surveillance of methicillin-resistant Staphylococcus aureus (MRSA) bacteraemia in children in the UK and Ireland.. Arch Dis Child.

[9] Feil EJ, Nickerson EK, Chantratita N, Wuthiekanun V, Srisomang P (2008). Rapid detection of the pandemic methicillin-resistant Staphylococcus aureus clone st 239, a dominant strain in asian hospitals.. J Clin Microbiol.

[10] Harris SR, Feil EJ, Holden MT, Quail MA, Nickerson EK (2010). Evolution of MRSA during hospital transmission and intercontinental spread.. Science.

[11] Holden MT, Lindsay JA, Corton C, Quail MA, Cockfield JD (2010). Genome sequence of a recently emerged, highly transmissible, multi-antibiotic- and antiseptic-resistant variant of methicillin-resistant Staphylococcus aureus, sequence type 239 (tw).. J Bacteriol.

[12] Edgeworth J, Yadegarfar G, Pathak S, Batra R, Cockfield J (2007). An outbreak in an intensive care unit of a strain of methicillin-resistant Staphylococcus aureus sequence type 239 associated with an increased rate of vascular access device-related bacteremia.. Clinical Infect Dis.

[13] May RM, Gupta S, McLean AR (2001). Infectious disease dynamics: What characterizes a successful invader?. Philos Trans R Soc Lond B Biol Sci.

[14] Read JM, Keeling MJ (2003). Disease evolution on networks: the role of contact structure.. Proc Biol Sci.

[15] Batra R, Cooper B, Whiteley C, Patel A, Wyncoll D (2010). Efficacy and limitation of a chlorhexidine-based decolonization strategy in preventing transmission of methicillin-resistant Staphylococcus aureus in an intensive care unit.. Clinical Infect Dis.

[16] Anderson RM, May RM (1992). Infectious diseases of humans: Dynamics and Control.

[17] Heesterbeek JAP, Dietz K (1996). The concept of *R0* in epidemic theory.. Statist Neerlandica.

[18] Ball F, Donnelly P (1995). Strong approximations for epidemic models.. Stochastic Process Appl.

[19] Vynnycky E, White RW (2010). An introduction to infectious disease modelling.

[20] Diekmann O, Heesterbeek JAP (2000). Mathematical epidemiology of infectious diseases. Wiley Series in Mathematical and Computational Biology.

[21] Wallinga J, Teunis P (2004). Different epidemic curves for severe acute respiratory syndrome reveal similar impacts of control measures.. Am J Epidemiol.

[22] White LF, Pagano M (2008). A likelihood-based method for real-time estimation of the serial interval and reproductive number of an epidemic.. Stat Med.

[23] Griffin JT, Garske T, Ghani AC, Clarke PS (2011). Joint estimation of the basic reproduction number and generation time parameters for infectious disease outbreaks.. Biostatistics.

[24] Farrington M, Redpath C, Trundle C, Coomber S, Brown NM (1998). Winning the battle but losing the war: methicillin-resistant Staphylococcus aureus (MRSA) infection at a teaching hospital.. QJM.

[25] Cooper B, Medley G, Stone S, Kibbler C, Cookson B (2004). Methicillin-resistant Staphylococcus aureus in hospitals and the community: stealth dynamics and control catastrophes.. Proc Natl Acad Sci U S A.

[26] Grundmann H, Hellriegel B (2006). Mathematical modelling: a tool for hospital infection control.. Lancet Infect Dis.

[27] Cooper B, Lipsitch M (2004). The analysis of hospital infection data using hidden Markov models.. Biostatistics.

[28] McBryde E, Pettitt A, McElwain D (2007). A stochastic mathematical model of methicillin resistant Staphylococcus aureus transmission in an intensive care unit: predicting the impact of interventions.. J Theor Biol.

[29] Forrester M, Pettitt A, Gibson G (2007). Bayesian inference of hospital-acquired infectious diseases and control measures given imperfect surveillance data.. Biostatistics.

[30] Bootsma M, Bonten M, Nijssen S, Fluit A, Diekmann O (2007). An algorithm to estimate the importance of bacterial acquisition routes in hospital settings.. Am J Epidemiol.

[31] Cooper B, Medley G, Bradley S, Scott G (2008). An augmented data method for the analysis of nosocomial infection data.. Am J Epidemiol.

[32] Kypraios T, O'Neill P, Huang S, Rifas-Shiman S, Cooper B (2010). Assessing the role of undetected colonization and isolation precautions in reducing methicillin-resistant Staphylococcus aureus transmission in intensive care units.. BMC Infect Dis.

[33] Haydon DT, Chase-Topping M, Shaw DJ, Matthews L, Friar JK (2003). The construction and analysis of epidemic trees with reference to the 2001 UK foot–and–mouth outbreak.. Proc Biol Sci.

[34] Cauchemez S, Bolle P, Donnelly C, Ferguson N, Thomas G (2006). Real-time estimates in early detection of SARS.. Emerg Infect Dis.

[35] Fraser C (2007). Estimating individual and household reproduction numbers in an emerging epidemic.. PLoS One.

[36] Kenah E, Lipsitch M, Robins JM (2008). Generation interval contraction and epidemic data analysis.. Math Biosci.

[37] Barnett AG, Batra R, Graves N, Edgeworth J, Robotham J (2009). Using a longitudinal model to estimate the effect of methicillin-resistant Staphylococcus aureus infection on length of stay in an intensive care unit.. Am J Epidemiol.

[38] Casewell MW (1995). New threats to the control of methicillin-resistant Staphylococcus aureus.. J Hosp Infect.

[39] Chambers HF, Deleo FR (2009). Waves of resistance: Staphylococcus aureus in the antibiotic era.. Nat Rev Microbiol.

[40] Bootsma MC, Wassenberg MW, Trapman P, Bonten MJ (2011). The nosocomial transmission rate of animal-associated st398 meticillin-resistant Staphylococcus aureus.. J R Soc Interface.

[41] Trapman P, Bootsma MC (2009). A useful relationship between epidemiology and queueing theory: the distribution of the number of infectives at the moment of the first detection.. Math Biosci.

[42] Wolkewitz M, Dettenkofer M, Bertz H, Schumacher M, Huebner J (2008). Statistical epidemic modeling with hospital outbreak data.. Stat Med.

[43] Mortimer EA, Wolinsky E, Gonzaga AJ, Rammelkamp CH (1966). Role of airborne transmission in staphylococcal infections.. Br Med J.

[44] Merrer J, Santoli F, Appéré de Vecchi C, Tran B, De Jonghe B (2000). Colonization pressure and risk of acquisition of methicillin-resistant Staphylococcus aureus in a medical intensive care unit.. Infect Control Hosp Epidemiol.

[45] Cauchemez S, Temime L, Valleron AJ, Varon E, Thomas G (2006). S. pneumoniae transmis- sion according to inclusion in conjugate vaccines: Bayesian analysis of a longitudinal follow-up in schools.. BMC Infect Dis.

[46] Coen P, Wilks M, Dall'antonia M, Millar M (2006). Detection of multiple-strain carriers: the value of re-sampling.. J Theor Biol.

[47] Batra R, Eziefula A, Wyncoll D, Edgeworth J (2008). Throat and rectal swabs may have an important role in MRSA screening of critically ill patients.. Intensive Care Med.

[48] Shinefield HR, Ribble JC, Boris M, Eichenwald HF, Aly R (1974). Bacterial interference between strains of S. aureus.. Ann N Y Acad Sci.

[49] Nelder JA, Mead R (1965). A simplex method for function minimization.. The Computer Journal.

