# Supplementary material for: Quantifying Type-Specific Reproduction Numbers for Nosocomial Pathogens: Evidence for Heightened Transmission of an Asian Sequence Type 239 MRSA Clone
Source: PLoS Comput Biol. 2012 Apr 12;8(4):e1002454. doi: 10.1371/journal.pcbi.1002454 (PMC3325179; doi:10.1371/journal.pcbi.1002454)
Supplement: Figure S1 — Single admission reproduction numbers ( ) estimated using method 2. Estimates (95% CIs) of the ward-level reproduction number, , according to study phase, MRSA type and ward obtained using method 2 and assuming complete bacterial interference and no interaction between ICU 1 and ICU 2. (PDF) [file pcbi.1002454.s001.pdf]

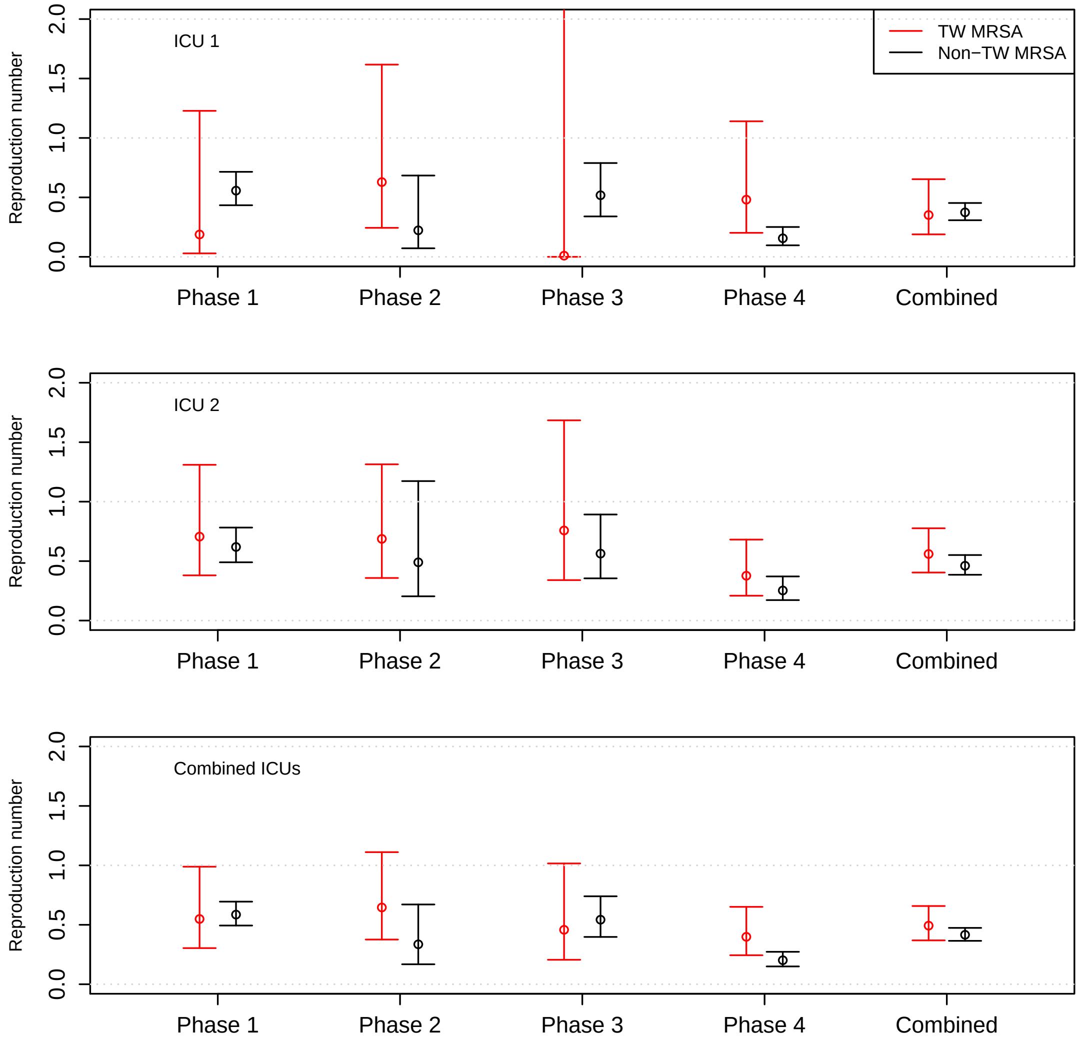

Figure S1. Single admission reproduction numbers (R_a) estimated using method 2. Estimates (95% CIs) of the ward-level reproduction number, R_a, according to study phase, MRSA type and ward obtained using method 2 and assuming complete bacterial interference and no interaction between ICU 1 and ICU 2.
