# Supplementary material for: Quantifying Type-Specific Reproduction Numbers for Nosocomial Pathogens: Evidence for Heightened Transmission of an Asian Sequence Type 239 MRSA Clone
Source: PLoS Comput Biol. 2012 Apr 12;8(4):e1002454. doi: 10.1371/journal.pcbi.1002454 (PMC3325179; doi:10.1371/journal.pcbi.1002454)
Supplement: Protocol S1 — Protocol for defining MRSA importation and acquisition events. (PDF) [file pcbi.1002454.s002.pdf]

Protocol for defining MRSA importation and acquisition events

Under the baseline assumption used in the main text each patient episode (i.e. uninterrupted period of stay) in an ICU is associated with either an MRSA importation event, an MRSA acquisition event, or no MRSA event according to the following definitions:

MRSA importation events are defined as events where one or more of the following criteria holds:

1. MRSA was recovered from a patient isolate which was taken within 48 hours of the patient being admitted to the ICU.
2. MRSA was recovered from a patient isolate and no negative MRSA screening swab was taken from the patient on any earlier day during the same ICU episode.
3. MRSA was recovered from a patient isolate taken fewer than 91 days before the patient was admitted to the ICU.

MRSA acquisition events are defined as events where either of the following criteria holds:

1. MRSA was recovered from the patient during the current ICU episode, but this episode did not meet the above criteria for an MRSA importation event.
2. MRSA was recovered from the patient within two days of ICU discharge and the episode did not meet the above criteria for an MRSA importation event.

All other ICU episodes were associated with no MRSA events. In these definitions a screening swab refers to a routine swab of the nose and any additional potential MRSA carriage sites that were swabbed at the same time (usually the axillae and perineum, and from November 2004 also the rectum and throat). Patient isolate refers to any clinical isolate taken directly from a patient (including blood, sputum, pus, pleural fluid, wound swab, line tip swab, drain fluid, skin swab) and screening swabs. The above criteria were applied by the infection control team throughout the course of the study period, and retrospectively checked for consistency by comparison with a database of all swabs and clinical isolates taken while patients were in one of the ICUs during the study period. Since the day but not the time of ICU admission and of clinical isolates were recorded in the database, it was assumed that MRSA isolates taken either one or two days after ICU admission were taken within 48 hours of admission. These checks led to changes in the MRSA status of 50 out of 4570 (1.1%) patient episodes and account for the slight discrepancies in the number of acquisitions reported here and elsewhere [1].

For the Method 2 analyses in the main text an assumed time of MRSA acquisition was also required. When the MRSA acquisition was detected by an MRSA positive isolate taken in the ICU we assumed the acquisition event to have occurred on the day the first MRSA positive isolate was taken. When MRSA acquisition was detected by an isolate immediately following ICU discharge, the acquisition was assumed to have occurred during the last day of stay in the ICU.

Sensitivity Analyses

Two sets of different assumptions about acquisition events and times were made so that we could test the sensitivity of our results to these assumptions. Sensitivity analysis 1 (SA1) used the above baseline definitions of acquisition and importation except that patient episodes where the first MRSA positive isolate was taken at least 24 hours after admission were also classified as MRSA acquisitions provided there was an MRSA negative screening swab taken on an earlier day from the same patient episode. Sensitivity analysis 2 (SA2) used the above baseline definitions of acquisition and importation but assumed acquisition events to have occurred two days before the first MRSA positive isolate was taken.

References

1. Batra R, Cooper B, Whiteley C, Patel A, Wyncoll D, et al. (2010) Efficacy and limitation of a chlorhexidine-based decolonization strategy in preventing transmission of methicillin-resistant Staphylococcus aureus in an intensive care unit. Clinical Infect Dis 50: 210–217.
