# Supplementary material for: Quantifying Type-Specific Reproduction Numbers for Nosocomial Pathogens: Evidence for Heightened Transmission of an Asian Sequence Type 239 MRSA Clone
Source: PLoS Comput Biol. 2012 Apr 12;8(4):e1002454. doi: 10.1371/journal.pcbi.1002454 (PMC3325179; doi:10.1371/journal.pcbi.1002454)
Supplement: Table S1 — TW and non-TW MRSA importation and acquisition events under different assumptions. The baseline assumption classifies all episodes where MRSA was recovered from an isolate taken within 48 hours of admission as importations. The SA1 assumption uses a 24 hour cutoff instead. See protocol S1 in supporting material for full details of baseline and SA1 assumptions. (PDF) [file pcbi.1002454.s003.pdf]

Table S1. TW and non-TW MRSA importation and acquisition events under different assumptions

	ICU 1				ICU 2			
	Importations		Acquisitions		Importations		Acquisitions	
	TW	Non-TW	TW	Non-TW	TW	Non-TW	TW	Non-TW
Baseline	12	270	23	109	25	260	43	123
SA1	11	268	24	111	24	256	44	127

The baseline assumption classifies all episodes where MRSA was recovered from an isolate taken within 48 hours of admission as importations. The SA1 assumption uses a 24 hour cutoff instead. See protocol in supporting material for full details of baseline and SA1 assumptions.
