# Supplementary material for: Quantifying Type-Specific Reproduction Numbers for Nosocomial Pathogens: Evidence for Heightened Transmission of an Asian Sequence Type 239 MRSA Clone
Source: PLoS Comput Biol. 2012 Apr 12;8(4):e1002454. doi: 10.1371/journal.pcbi.1002454 (PMC3325179; doi:10.1371/journal.pcbi.1002454)
Supplement: Table S2 — Estimated ward-level reproduction numbers (s.e.) for TW and non-TW MRSA clones under alternative assumptions. Phase-specific estimates of ward-level reproduction numbers for TW MRSA and Non-TW MRSA derived using Method 1 under baseline assumptions with perfect ward coupling (applies to combined ICU estimates only) and under SA1 assumptions (see protocol S1 in supporting material for details of baseline and SA1 assumptions). (PDF) [file pcbi.1002454.s004.pdf]

Table S2. Estimated ward-level reproduction numbers (s.e.) for TW and non-TW MRSA clones under alternative assumptions

		Phases			
		1	2	3	4
Baseline assumptions but with perfect ward coupling					
Combined ICUs	TW MRSA	0.71 (0.17)	0.50 (0.09)	0.56 (0.17)	0.64 (0.15)
Combined ICUs	Non-TW MRSA	0.38 (0.02)	0.20 (0.03)	0.47 (0.08)	0.15 (0.02)
Assumption SA1					
ICU1	TW MRSA	0.14 (0.10)	0.65 (0.17)	0.13 (0.08)	0.54 (0.22)
ICU1	Non-TW MRSA	0.37 (0.03)	0.31 (0.08)	0.41 (0.07)	0.14 (0.03)
ICU2	TW MRSA	0.73 (0.19)	0.41 (0.11)	0.58 (0.22)	0.58 (0.17)
ICU2	Non-TW MRSA	0.42 (0.04)	0.21 (0.06)	0.54 (0.16)	0.17 (0.03)
Combined ICUs	TW MRSA	0.69 (0.19)	0.51 (0.10)	0.58 (0.15)	0.63 (0.14)
Combined ICUs	Non-TW MRSA	0.38 (0.03)	0.21 (0.04)	0.46 (0.08)	0.15 (0.02)

Phase-specific estimates of ward-level reproduction numbers for TW MRSA and Non-TW MRSA derived using Method 1 under baseline assumption assuming perfect ward coupling (applies to combined ICU estimates only) and under SA1 assumptions (see protocol in supporting matieral for details of baseline and SA1 assumptions).
