# Supplementary material for: Quantifying Type-Specific Reproduction Numbers for Nosocomial Pathogens: Evidence for Heightened Transmission of an Asian Sequence Type 239 MRSA Clone
Source: PLoS Comput Biol. 2012 Apr 12;8(4):e1002454. doi: 10.1371/journal.pcbi.1002454 (PMC3325179; doi:10.1371/journal.pcbi.1002454)
Supplement: Table S3 — estimates for TW and non-TW combined under SA1 and SA2 assumptions. Sensitivity analysis for phase-specific estimates for , the daily probability of a susceptible patient acquiring MRSA from an MRSA positive patient in the same ward, for ICU 1 and ICU 2 (without distinguishing between TW and non-TW strains). In the Combined row, the estimates are constrained to be the same in both wards, and the All phases column constrains the estimates to be the same in the four phases. See protocol S1 in supporting material for details of the SA1 and SA2 assumptions used in the sensitivity analyses. 1 P-values test the null hypothesis that transmission does not vary between study phase (likelihood ratio test, df = 3). 2 P-values test the null hypothesis that transmission in the current phase does not differ between wards (likelihood ratio test, df = 1). (PDF) [file pcbi.1002454.s005.pdf]

Table S3: q estimates for TW and non-TW combined under SA1 and SA2 assumptions

	Phases					P-value[1]
	1	2	3	4	All phases	
SA1 Assumptions						
ICU1	0.0032 (0.0025, 0.0041)	0.0033 (0.0019, 0.0059)	0.0032 (0.0021, 0.0046)	0.0012 (0.0008, 0.0018)	0.0024 (0.0020, 0.0029)	< 0.0001
ICU2	0.0037 (0.0030, 0.0046)	0.0036 (0.0022, 0.0059)	0.0038 (0.0027, 0.0056)	0.0016 (0.0012, 0.0022)	0.0029 (0.0025, 0.0033)	< 0.0001
Combined	0.0035 (0.0030, 0.0041)	0.0035 (0.0024, 0.0051)	0.0035 (0.0027, 0.0046)	0.0014 (0.0011, 0.0018)	0.0027 (0.0024, 0.0030)	< 0.0001
P-value[2]	0.15	0.37	0.82	0.45	0.26	
SA2 Assumptions						
ICU1	0.0032 (0.0025, 0.0041)	0.0029 (0.0016, 0.0053)	0.0032 (0.0022, 0.0047)	0.0012 (0.0008, 0.0018)	0.0024 (0.0020, 0.0028)	< 0.0001
ICU2	0.0036 (0.0029, 0.0044)	0.0028 (0.0016, 0.0049)	0.0037 (0.0026, 0.0054)	0.0015 (0.0011, 0.0021)	0.0027 (0.0023, 0.0031)	< 0.0001
Combined	0.0034 (0.0029, 0.0040)	0.0029 (0.0019, 0.0043)	0.0034 (0.0026, 0.0045)	0.0014 (0.0011, 0.0018)	0.0025 (0.0023, 0.0029)	< 0.0001
P-value[2]	0.34	0.50	0.89	0.58	0.45	

Sensitivity analysis for phase-specific estimates for q, the daily probability of a susceptible patient acquiring MRSA from an MRSA positive patient in the same ward, for ICU 1 and ICU 2 (without distinguishing between TW and non-TW strains). In the *Combined* row, the estimates are constrained to be the same in both wards, and the *All phases* column constrains the estimates to be the same in the four phases. See protocol in supporting material for details of the SA1 and SA2 assumptions used in the sensitivity analyses.

1 P-values test the null hypothesis that transmission does not vary between study phase (likelihood ratio test, df=3).

2 P-values test the null hypothesis that transmission in the current phase does not differ between wards (likelihood ratio test, df=1).
