# Supplementary material for: Quantifying Type-Specific Reproduction Numbers for Nosocomial Pathogens: Evidence for Heightened Transmission of an Asian Sequence Type 239 MRSA Clone
Source: PLoS Comput Biol. 2012 Apr 12;8(4):e1002454. doi: 10.1371/journal.pcbi.1002454 (PMC3325179; doi:10.1371/journal.pcbi.1002454)
Supplement: Table S4 — Estimates of the daily transmission probability (q) from one exposed to one susceptible patient under SA1 and SA2 assumptions. Estimates of the daily transmission probability (q) from one exposed to one susceptible patient under assumptions SA1 and SA2. See protocol S1 in supporting material for details of the SA1 and SA2 assumptions used in thesse sensitivity analyses. 1. P-values test the null hypothesis that transmission varies between study phases but not MRSA types against the alternative that it varies between study phases and MRSA types (likelihood ratio test, df = 4). 2 P-values test the null hypothesis that transmission in the study phase does not differ between TW and Non-TW MRSA using combined data from both wards (likelihood ratio test, df = 1). (PDF) [file pcbi.1002454.s006.pdf]

Table S4: Estimates of the daily transmission probability (q) from one exposed to one susceptible patient under SA1 and SA2 assumptions

		Phases					P-value[1]
		1	2	3	4	All phases	
SA1 Assumptions							
ICU1	TW MRSA	0.0009(0.0001,0.0068)	0.0042(0.0018,0.0101)	0.0000(0.0000,0.2500)	0.0026(0.0011,0.0063)	0.0022(0.0012,0.0039)	0.01
	Non-TW MRSA	0.0032(0.0025,0.0041)	0.0012(0.0004,0.0038)	0.0029(0.0019,0.0045)	0.0009(0.0005,0.0014)	0.0021(0.0017,0.0026)	
ICU2	TW MRSA	0.0038(0.0021,0.0071)	0.0041(0.0022,0.0076)	0.0041(0.0018,0.0091)	0.0021(0.0011,0.0037)	0.0031(0.0023,0.0043)	0.70
	Non-TW MRSA	0.0035(0.0028,0.0044)	0.0026(0.0011,0.0063)	0.0032(0.0021,0.0050)	0.0014(0.0009,0.0020)	0.0026(0.0022,0.0031)	
Both	TW MRSA	0.0030(0.0017,0.0054)	0.0041(0.0025,0.0068)	0.0025(0.0011,0.0056)	0.0022(0.0013,0.0036)	0.0028(0.0021,0.0038)	< 0.0001
	Non-TW MRSA	0.0033(0.0028,0.0039)	0.0019(0.0009,0.0037)	0.0031(0.0023,0.0042)	0.0011(0.0008,0.0015)	0.0023(0.0021,0.0027)	
P-value[2]		0.75	0.06	0.64	0.03	0.24	
SA2 Assumptions							
ICU1	TW MRSA	0.0014(0.0003,0.0069)	0.0047(0.0022,0.0103)	0.0017(0.0004,0.0078)	0.0027(0.0012,0.0062)	0.0022(0.0012,0.0039)	0.15
	Non-TW MRSA	0.0032(0.0025,0.0041)	0.0022(0.0010,0.0051)	0.0032(0.0022,0.0048)	0.0010(0.0006,0.0015)	0.0022(0.0018,0.0026)	
ICU2	TW MRSA	0.0041(0.0023,0.0074)	0.0032(0.0016,0.0064)	0.0045(0.0021,0.0093)	0.0020(0.0011,0.0036)	0.0030(0.0022,0.0042)	0.42
	Non-TW MRSA	0.0033(0.0026,0.0041)	0.0016(0.0005,0.0049)	0.0031(0.0020,0.0049)	0.0012(0.0008,0.0018)	0.0024(0.0020,0.0029)	
Both	TW MRSA	0.0032(0.0018,0.0056)	0.0034(0.0019,0.0058)	0.0032(0.0016,0.0063)	0.0021(0.0013,0.0034)	0.0028(0.0021,0.0037)	< 0.0001
	Non-TW MRSA	0.0032(0.0027,0.0038)	0.0016(0.0008,0.0034)	0.0031(0.0023,0.0041)	0.0011(0.0008,0.0015)	0.0023(0.0020,0.0026)	
P-value[2]		1	0.10	1	0.03	0.22	

Estimates of the daily transmission probability (q) from one exposed to one susceptible patient under assumptions SA1 and SA2. See protocol in supporting material for details of the SA1 and SA2 assumptions used in thesse sensitivity analyses.
1. P-values test the null hypothesis that transmission varies between study phases but not MRSA types against the alternative that it varies between study phases and MRSA types (likelihood ratio test, df=4).
2 P-values test the null hypothesis that transmission in the study phase does not differ between TW and Non-TW MRSA using combined data from both wards (likelihood ratio test, df=1).
