# Supplementary material for: Quantifying Type-Specific Reproduction Numbers for Nosocomial Pathogens: Evidence for Heightened Transmission of an Asian Sequence Type 239 MRSA Clone
Source: PLoS Comput Biol. 2012 Apr 12;8(4):e1002454. doi: 10.1371/journal.pcbi.1002454 (PMC3325179; doi:10.1371/journal.pcbi.1002454)
Supplement: Table S5 — Estimates of the daily transmission probability (q) from one exposed to one susceptible patient and from background transmission sources. ‘Patient to patient’ estimates corresponds to the daily transmission probability (q) from one exposed to one susceptible patient. Background estimates corresponds to the daily probability of acquisition from background sources (such as environmental contamination). This probability is assumed to remain constant within each phase for each of the two MRSA types. In some cases confidence intervals could not be estimated for numerical reasons, while in others the very wide confidence intervals indicate that parameters are weakly identifiable. For both ICUs we compared the model with background and patient-to-patient transmission (with both phase and MRSA-type specific parameters) with nested models with only background transmission (but still with both phase and MRSA-type specific parameters) using a likelihood ratio test based on the chi-squared distribution with eight degrees of freedom. The results gave strong evidence to prefer the more complex model in the case of ICU2 (p = 0.008), but no evidence to prefer it in the case of ICU 1 (p = 0.70). (PDF) [file pcbi.1002454.s007.pdf]

**Table S5: Estimates of the daily transmission probability (q) from one exposed to one susceptible patient and from background transmission sources.**

| | | Phases | | | |
|---|---|---|---|---|---|
| | | 1 | 2 | 3 | 4 |
| ICU1 | | | | | |
| Patient to patient | TW MRSA | 0.0000(–,–) | 0.0003(0.0000,0.0808) | 0.0001(0.0000,0.2229) | 0.0022(0.0007,0.0064) |
| Background | TW MRSA | 0.0007(0.0003,0.0019) | 0.0070(0.0032,0.0153) | 0.0024(0.0009,0.0063) | 0.0005(0.0002,0.0014) |
| Patient to patient | Non-TW MRSA | 0.0003(0.0000,0.4661) | 0.0000(0.0000,1.0000) | 0.0012(0.0002,0.0089) | 0.0000(0.0000,0.3752) |
| Background | Non-TW MRSA | 0.0103(0.0044,0.0242) | 0.0039(0.0014,0.108) | 0.0077(0.0020,0.00298) | 0.0022(0.0013,0.0038) |
| ICU2 | | | | | |
| Patient to patient | TW MRSA | 0.0035(0.0017,0.0070) | 0.0037(0.0019,0.0070) | 0.0004(–,–) | 0.0019(0.0010,0.0036) |
| Background | TW MRSA | 0.0007(0.0002,0.0022) | 0.0000(–,–) | 0.0042(0.0024,0.0074) | 0.0002(0.0000,0.0014) |
| Patient to patient | Non-TW MRSA | 0.0001(0.0000,0.0015) | 0.0001(0.0000,0.37) | 0.0006(0.0000,0.0767) | 0.0001(0.0000,0.0391) |
| Background | Non-TW MRSA | 0.0120(0.0089,0.0161) | 0.0064(0.0025,0.0163) | 0.0077(0.0023,0.0259) | 0.0032(0.0020,0.0054) |

‘Patient to patient’ estimates corresponds to of the daily transmission probability (q) from one exposed to one susceptible patient. ‘Background’ estimates corresponds to the daily probability of acquisition from background sources (such as environmental contamination). This probability is assumed to remain constant within each phase for each of the two MRSA types. In some cases confidence intervals could not be estimated for numerical reasons, while in others the very wide confidence intervals are indicate that parameters are weakly identifiable. For both ICUs we compared the model with background and patient-to-patient transmission (with both phase and MRSA-type specific parameters) with nested models with only background transmission (but still with both phase and MRSA-type specific parameters) using a likelihood ratio test based on the chi-squared distribution with eight degrees of freedom. The results gave strong evidence to prefer the more complex model in the case of ICU2 (p=0.008), but no evidence to prefer it in the case of ICU 1 (p=0.70).
